# QRTEngine: An easy solution for running online reaction time experiments using Qualtrics

**DOI:** 10.3758/s13428-014-0530-7

**Published:** 2014-11-19

**Authors:** Jonathan S. Barnhoorn, Erwin Haasnoot, Bruno R. Bocanegra, Henk van Steenbergen

**Affiliations:** 1 Institute of Psychology, Leiden University, Wassenaarseweg 52, Leiden, 2333 AK The Netherlands; 2 Department of Cognitive Psychology and Ergonomics, Faculty of Behavioral, Management and Social Sciences, University of Twente, Enschede, The Netherlands; 3 University of Sheffield, Sheffield, UK; 4 Leiden Institute for Brain and Cognition, Leiden University, Leiden, The Netherlands; 5 MIRA Research Institute, University of Twente, Enschede, The Netherlands

**Keywords:** Online experiments, Qualtrics, JavaScript, Amazon Mechanical Turk, Open-source

## Abstract

Performing online behavioral research is gaining increased popularity among researchers in psychological and cognitive science. However, the currently available methods for conducting online reaction time experiments are often complicated and typically require advanced technical skills. In this article, we introduce the Qualtrics Reaction Time Engine (QRTEngine), an open-source JavaScript engine that can be embedded in the online survey development environment Qualtrics. The QRTEngine can be used to easily develop browser-based online reaction time experiments with accurate timing within current browser capabilities, and it requires only minimal programming skills. After introducing the QRTEngine, we briefly discuss how to create and distribute a Stroop task. Next, we describe a study in which we investigated the timing accuracy of the engine under different processor loads using external chronometry. Finally, we show that the QRTEngine can be used to reproduce classic behavioral effects in three reaction time paradigms: a Stroop task, an attentional blink task, and a masked-priming task. These findings demonstrate that QRTEngine can be used as a tool for conducting online behavioral research even when this requires accurate stimulus presentation times.

**Electronic supplementary material:**

The online version of this article (doi:10.3758/s13428-014-0530-7) contains supplementary material, which is available to authorized users.

In the past decade, psychologists have showed increasing interest in conducting research via the Internet. Through online labor markets such as Amazon’s Mechanical Turk (AMT), high numbers of participants can be tested in a short amount of time and at low cost. Recently, concerns about the quality of data gathered through AMT have been addressed, and multiple studies have now shown that data gathered using AMT are reliable and comparable to data obtained in the lab (Behrend, Sharek, Meade, & Wiebe, 2011; Buhrmester, Kwang, & Gosling, 2011; Paolacci, Chandler, & Ipeirotis, 2010). These validation studies, like most previous research conducted via AMT, were survey-based. Conducting online experiments that rely on precise recording of reaction times (RTs) is much more difficult, although prior work using basic HTML and JavaScript has successfully replicated a number of RT tasks online (Crump, McDonnell, & Gureckis, 2013).

A number of methods have recently been developed to help psychological and cognitive scientists program RT experiments for the Internet. Currently available solutions include Tatool (von Bastian, Locher, & Ruflin, 2013), WebExp (Keller, Gunasekharan, Mayo, & Corley, 2009), and ScriptingRT (Schubert, Murteira, Collins, & Lopes, 2013), which have all been developed with the aim of providing precise timing 1. These libraries can be used to produce RT experiments that are cross-platform and cross-browser compatible. To run an experiment in Tatool or WebExp, participants need to have Java installed on their computer, whereas ScriptingRT is based on Adobe Flash. Importantly, in order to create RT experiments, these solutions typically still require substantial programming skill, and the researcher needs to host a Web server to publish the experiment. Furthermore, since these methods require specialized software or plugins, they do not meet the AMT constraint that the participant may not be required to install additional software.

## Introducing the QRTEngine

Here we present an alternative method for conducting online RT experiments that is based on the existing online survey development environment Qualtrics. The Qualtrics Reaction Time Engine (QRTEngine) is different from the previously described methods in a number of ways: It is hosted on the Qualtrics server; it does not require specialized software or browser plugins; it is more precise than the previously described JavaScript method; and because it is embedded in the user-friendly Qualtrics interface, most of the basic functionality can be used by researchers who are not experienced programmers.

Using the basic Qualtrics environment, one can set up sophisticated surveys, publish them, and collect the results. Typically, a survey is created by adding questions (which can consist of just text and/or images as well) that are organized in particular blocks. In order to set up an RT experiment in Qualtrics, the QRTEngine modifies the way that questions in a block are presented. When several questions are set up without a page break, Qualtrics would normally present them together on the page. When the QRTEngine is included, however, questions are presented one screen after the other, allowing for control over the timing of each screen. Furthermore, the QRTEngine provides a way (or API) for conveniently adding keyboard responses, setting up conditional and/or delayed display of screens, and setting more advanced properties. The QRTEngine itself consists of a piece of JavaScript code, called the “engine,” that can be easily included in any Qualtrics survey. Question screens are created using the standard Qualtrics visual editor, whereas the properties of the RT task screens can be set by copying and pasting and adapting standard JavaScript snippets to each question. The Qualtrics “Loop & Merge” functionality is used to set the list of trials and their specific properties in a convenient way. To control timing-related aspects, the QRTEngine uses a recent HTML5 method, allowing for more accurate results than provided by comparable older JavaScript-based methods.

The QRTEngine was developed with researchers in mind who are not experienced programmers but who have used experiment-builder applications such as E-Prime or OpenSesame before (Mathôt, Schreij, & Theeuwes, 2012; Schneider, Eschman, & Zuccolotto, 2002). Like most experiment-builder methods, the QRTEngine provides increasingly sophisticated functionality for increasingly complex programming. For users without a background in programming, we expect that the example tasks and JavaScript snippets, provided at www.qrtengine.com, will be sufficient to help them program simple paradigms themselves. For researchers who already have substantial programming skills, we think the QRTEngine can provide a method to speed up development: The Qualtrics environment provides many tools that make the building process more efficient, and the QRTEngine conveniently wraps up the complex calls that are necessary to implement the timing and dynamic properties of an experiment. Furthermore, Qualtrics provides some handy participant management features. The QRTEngine is published open-source under the Apache 2.0 license, meaning that experienced programmers are welcome to consult our method when programming solutions from scratch. When doing this, it is important to realize that the QRTEngine is not a standalone library and has dependencies on Qualtrics and the Prototype Framework (which is already present in any Qualtrics survey). As we will demonstrate in this article, we think that the QRTEngine is particularly useful for running plugin-free browser-based RT experiments that present text and pictures, provided that accurate timing and a stable intertrial interval are not critical.

## Recommendations for online experimentation

Before using the QRTEngine, a researcher should carefully consider whether performing an online experiment is the right choice in the first place, and whether the QRTEngine is the optimal method for the planned study. The pros and cons of online research in general have already been discussed in some excellent previous publications (Behrend et al., 2011; Buhrmester et al., 2011; Mason & Suri, 2012; Paolacci et al., 2010). When focusing on RT tasks specifically, some additional limitations should be taken into consideration as well. The computer systems that participants use vary widely, and the error involved in measuring RT data and ensuring precise display durations on a given system is largely unknown. In addition, keyboards have different sampling rates (depending on both hardware and the operating system and browser combination), and monitor refresh rates also vary. Furthermore, continuously changing operating systems and Web browsers both contribute to the uncertainty in precision. When users run multiple applications on their computer, this can also affect timing randomly. Essentially, there are many sources of potential timing errors. These errors typically are expected to be random across subjects and conditions, and therefore, multitrial designs and collecting time audit information are necessary so that experimenters can filter out extreme timing errors in order to still obtain reliable results (Ulrich & Giray, 1989). Hence, we recommend conducting online RT experiments only when it is acceptable to test more participants in order to compensate for the noise introduced when using online acquisition methods.

## Using the QRTEngine

A detailed introduction to using the QRTEngine is provided in the supplementary material as a step-by-step tutorial on how to create a simple Stroop task (MacLeod, 1991; Stroop, 1935). In order to build this task, one only needs a computer with an Internet connection and a Qualtrics account. In this article, we provide a concise overview of the development process of this same Stroop task.

**Fig. 1:**
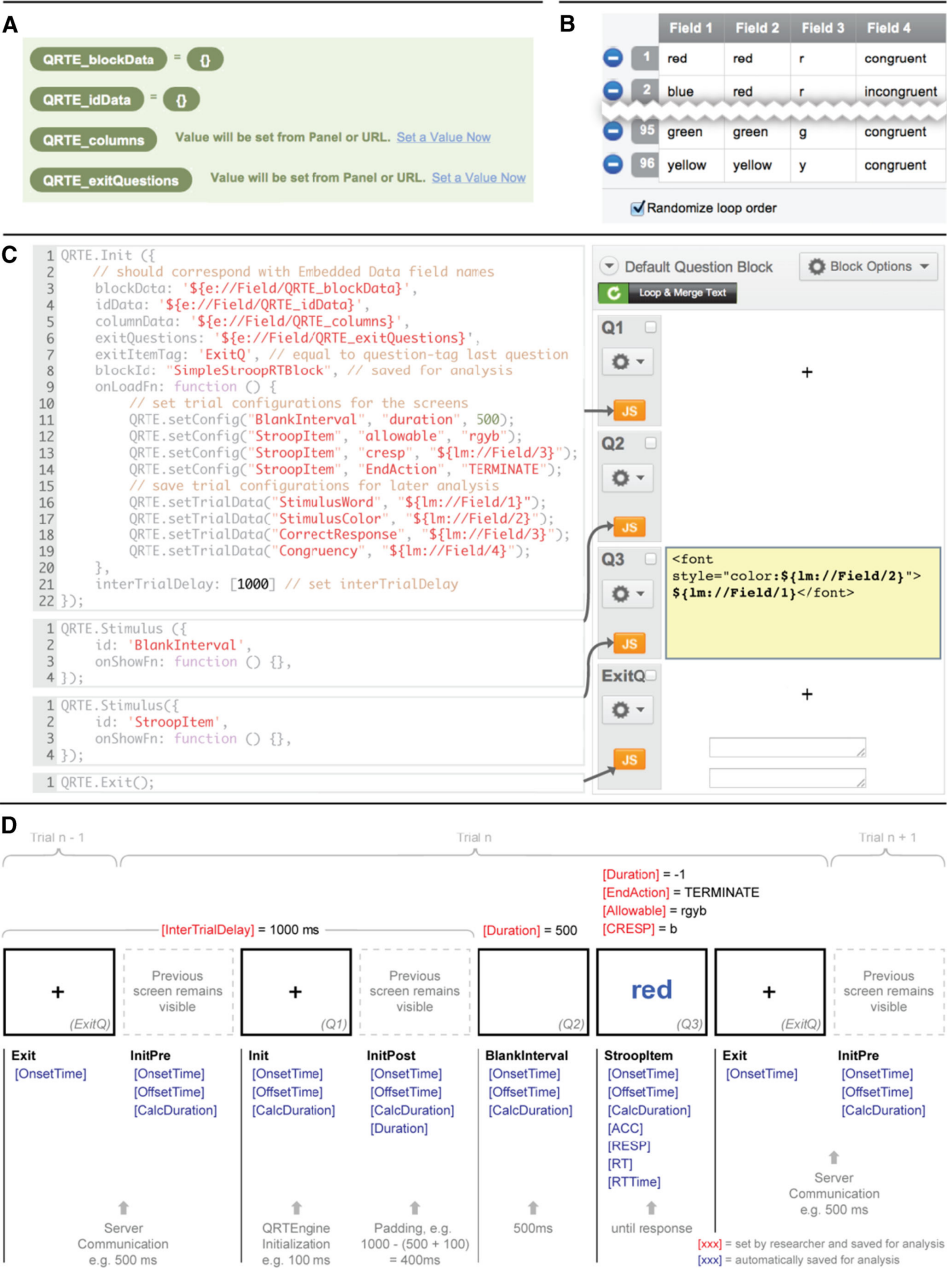
(**a**) Screenshot of what the embedded data overview should look like for the Stroop reaction time (RT) task. (**b**) Screenshot of the Loop & Merge list. In the Stroop RT task, 96 trials will be displayed, and four columns are needed to define the variable content for each trial. (**c**) Screenshot of the question block along with the JavaScript for each question. Each question represents a screen in the task. (**d**) Schematic overview of a trial in the Stroop RT task

The QRTEngine is included into a survey by pasting the JavaScript code (available via www.qrtengine.com) in the survey’s header. Then, a number of embedded data fields need to be created that are used by the engine during the runtime of the experiment (see Fig. 1a). Finally, the layout needs to be selected; we recommend using the standard Qualtrics “Minimal” layout.

The QRTEngine works by looping a question block according to a predefined list of trial properties that is specified under the “Loop & Merge” functionality. In the case of a Stroop task, the Loop & Merge list specifies the correct response, the color of the word, and the word (see Fig. 1b). Each question in the question block is displayed as a screen in the task. One can include as many screens and responses in one trial as desired. It is also possible to set a screen to be displayed in the background through the whole trial—for example, a response key reminder. Furthermore, a screen showing accuracy feedback for a specified duration can also be added as an extra question. The timing and response configurations for a question are defined in the JavaScript code corresponding to that question (see Fig. 1c). After setting these configurations, the survey is finished and can be distributed.

The trial information, including the individual time audit information, is saved using a JSON representation within the standard Qualtrics wide data format. Saving the data in a format that can be directly imported into packages like SPSS or Excel is not possible, due to restrictions in the Qualtrics API. Therefore, we published the QRTEParser, a cross-platform Java program that is available on www.qrtengine.com and allows for converting the Qualtrics QRTEngine comma-separated value (CSV) file to a long format (where each row presents the data of a single trial). Standard packages such as SPSS and Excel can then be used to read the resulting CSV data file and perform data aggregation and statistics.

## Timing features of the QRTEngine

The QRTEngine has been developed with the goal of providing timing capabilities as accurate as possible within the limits of modern Web browser technology. In this section, we discuss which actions we took to achieve this goal.

### Logging of timing-related information

The QRTEngine uses an HTML5 feature called “requestAnimationFrame” (rAF) to synchronize the onset of stimuli with the refresh rate of the monitor. When using rAF, the QRTEngine is notified by the browser (through a high-priority function callback) no more than once every screen refresh that a screen refresh is taking place. Hence, every time that rAF notifies the QRTEngine, it can use a timer to determine whether the elapsed screen presentation time approaches the intended presentation time. In this way, the QRTEngine can estimate whether the last screen refresh of an intended screen duration is to be expected, and prepare the next screen accordingly. To correct for potential imprecision due to imperfect resolution of the timer, we recommend subtracting 5 ms from the intended duration. The rAF feature is currently supported in all modern browsers. In older browsers, rAF may not be available,^2^ in which case the QRTEngine falls back to the less precise setTimeout() method (a low-priority function callback) to control the duration of presentation.

When the duration for a screen has been set, the QRTEngine logs the following time audit information during runtime: Duration, which is the intended duration set by the researcher; OnsetTime and OffsetTime, which provide time stamps in milliseconds relative to Unix epoch; and CalculatedDuration, which represents the estimated actual duration of screen presentation, based on the difference between the OnsetTime and OffsetTime. The CalculatedDuration can thus be used to estimate how much the actual duration of presentation deviated from the intended duration. Figure 1d provides an overview of all of the information that is saved during the example Stroop task.

The time stamps are generated using one of two timers based on browser support. The most precise timer uses the JavaScript performance.now method, supported by all modern browsers except Safari.^3^ According to the W3C definition, this timer should be accurate to the microsecond, but is at least accurate to the millisecond. The performance.now timer is independent from the system clock, and thus is not subject to system clock adjustments or system clock skew (Mann, 2012). In older browsers, the QRTEngine falls back to the JavaScript date.now timer, which is less precise. The QRTEngine logs the availability of rAF and the type of timer under the “EngineType” attribute using the following labels (ordered from most to least precise): “native-highres,” when rAF and performance.now are both available; “native,” when rAF is available but only the date.now timer can be used; and “timer,” when setTimeout is used in combination with the date.now timer. Information from the “EngineType” attribute can be used to exclude participants whose browser did not support precise rAF and/or precise time stamps when an experiment is very sensitive to timing.

Providing exact statements about the precision of the time stamps and the timing of screen presentation is not possible, because multiple factors can influence this precision. For example, when running many animations in multiple tabs or when a laptop’s battery power is low, the performance.now timer resolution is decreased in some browsers in order to save CPU power. Furthermore, it may sometimes happen that for unknown reasons, the rAF function call skips a frame. In general, very high CPU and especially RAM load can be expected to be associated with decreased precision of the timers. To estimate the precision of screen durations with the QRTEngine during an experiment, we therefore performed a validation study using external chronometry.

### Validation using photosensitive diode

To investigate the accuracy of stimulus presentation timing using the QRTEngine, we conducted a timing validation study using a method similar to that reported by Simcox and Fiez (2014) to validate the timing accuracy of Adobe Flash. Accordingly, we presented stimuli under different CPU and RAM load conditions and compared the intended durations with the durations as measured by a photosensitive diode. We also compared the photodiode measurements to the durations logged by the QRTEngine in the CalculatedDuration attribute. The diode was placed on the computer screen on which the experiment was displayed and monitored via the line-in jack of a separate desktop computer running Windows Sound Recorder at a sampling rate of 44.1 kHz (this computer ran no other programs during recording). Offline analysis was performed in MATLAB and SPSS. Because of the variety of computer systems, operating systems, and Web browsers, testing every possible configuration is not possible. Therefore, we selected two configurations that reasonably represent typical configurations.

#### Method

The experimental survey was run on two systems. System 1 was a BTO laptop running Windows 7 Ultimate on a 2.5-GHz Intel i5 quad core processor with 8 GB of RAM. The experiment was conducted running Chrome 27 on a Targa CRT monitor running at 60 Hz. System 2 was a MacBook Pro running OSX 10.5.8 on a 2.4-GHz Intel Core 2 Duo processor with 4 GB of RAM. The experiment was conducted running Firefox 16.0.2 with the same Targa CRT monitor running at 60 Hz. Similar to the experiment reported by Simcox and Fiez (2014), both systems used Prime95 version 27.9 to manipulate CPU and RAM load in a controllable, predictable way (Woltman, 2012). The four load conditions were (1) low, in which only the browser was running; (2) medium, in which Prime95 ran a torture test using 50 % of the CPU; (3) high, in which Prime95 ran a torture test using 100 % of the CPU; and (4) maximum, in which the torture test was run using up almost all RAM, as well.

The survey that we created for the experiment consisted of a white screen on which a large black square was presented approximately 40 times for durations of 1, 2, 3, 4, 5, 6, 12, 30, and 60 frames (in that order, with each duration condition fully completed before the next condition). The interval between the presentations of two squares was set to 1,500 ms. The total duration of the experiment was about 12 min.

#### Results and discussion

In order to estimate the accuracy of the presentation timing, we compared the actual stimulus duration measured using the photosensitive diode with (1) the duration set by the experimenter and (2) the calculated duration logged in the QRTE data file. The mean absolute differences between these measures are displayed in Table 1 (for details, see the supplementary material).

**Table 1:** Differences between intended, actual, and logged durations in the timing validation study

Computer Load	Mean (ms)					
	| Intended Duration – Actual Duration |			| Logged Duration – Actual Duration |		
	System 1	System 2	Average	System 1	System 2	Average
Low	1.2	9.9	5.6	0.9	9.7	5.3
Med	2.2	10.9	6.6	1.8	9.6	5.7
High	1.5	9.5	5.5	1.4	10.1	5.8
Max	9.3	10.7	10.0	6.6	10.0	8.3
Average	3.5	10.2	6.9	2.7	9.9	6.3

System 1 = BTO laptop running Windows 7; System 2 = MacBook Pro running OSX 10.5.8

As Table 1 shows, the average deviation of both measures is around 6 ms in the low-load conditions. Only under conditions of maximum load was a substantial deterioration in performance observed in both systems, leading to an average deviation of around 10 ms. The results also indicate a difference in performance between the two systems. It may have been that this difference was due to the fact that the System 2 hardware and software were quite old (the operating system was released in 2007 and has lost support by current browsers). However, this difference in performance also illustrates a general caveat in online experimenting: The experimenter simply cannot know all hardware and other factors that will influence performance. Our findings show that the mean deviation between the intended duration of a stimulus and the actual deviation of a stimulus was small, and given the 60-Hz display rate used here, falls within the range of ± 1 display frame deviation (16.67 ms). A similar accuracy was observed for the calculated duration attribute that is logged in the QRTE data file. Researchers might use this attribute to get a reasonably reliable estimate of timing errors, which can be used to exclude trials or participants on an individual basis when accurate timing is critical. Apart from the mean deviation in milliseconds, we also analyzed the deviation between the photodiode measurement and the intended duration as expressed in the number of frames (16.67-ms units, in our test case). The results of these analyses are reported in Table 2. The results show that across the systems, including the low-, medium-, and high-load conditions, a timing accuracy of the intended duration within the range of ± 1 frame deviation was present in 97 % of the trials. In the supplementary material, we provide tables similar to Table 2 for the trials in which only one or two frames were presented.

**Table 2:** Percentages of observed deviations, in frames, between intended and actual durations in the timing validation study

	System 1				System 2			
	0	1	2	>2	0	1	2	>2
Low load	93.0 %	6.6 %	0.2 %	0.0 %	46.1 %	50.5 %	2.2 %	1.1 %
Med load	87.2 %	12.2 %	0.5 %	0.0 %	45.8 %	45.8 %	6.9 %	1.3 %
High load	91.9 %	7.2 %	0.8 %	0.0 %	49.1 %	46.3 %	3.8 %	0.5 %
Max load	59.1 %	32.7 %	3.6 %	4.4 %	48.6 %	42.2 %	7.7 %	1.3 %

System 1 = BTO laptop running Windows 7; System 2 = MacBook Pro running OSX 10.5.8

### Server communication delay

Another important timing-related aspect concerns the communication with the Qualtrics server. Because the implementation of a block of trials depends on the native Qualtrics Loop & Merge functionality, at the end of every trial the data that have been collected are automatically sent to the Qualtrics server. The speed of this server communication relies on factors such as the Internet connection of the participant and on the load of the Qualtrics server. The duration of the server communication time is variable and not under control of the researcher.

When setting up a QRTEngine survey, the researcher defines an intertrial delay (ITD), which is essentially the minimum delay between the end of the current and the beginning of the next trial (the intertrial interval), during which period server communication takes place. Please note that preloading (or caching) trials is not possible when using the Qualtrics environment. As is displayed in Fig. 1d, the ITD is the minimum time that will elapse between two consecutive trials set by the researcher. During the ITD, the last screen of trial *n* and the first screen of trial *n* + 1 are displayed, while in the meantime three processes take place. The first process, InitPre, takes care of the server communication during which the collected information is sent to the server and the data regarding the next trial are received from the server. When server communication is complete, the second process, called Init, starts, which initializes the next trial. The third process, InitPost, fills up the remaining ITD that was set by the researcher. If the combined duration of InitPre and Init exceeds the ITD, the duration of the InitPost is set to 0. The time stamps of all three processes are logged separately (see also Fig. 1d).

In the validation experiments, to be discussed in the next section, we measured the duration of the InitPre and Init processes. Because we anticipated that a slightly longer ITD for some participants would not negatively influence our results, we allowed MTurk workers to participate if their estimated server communication delay was lower than 2,000 ms.^4^ Across the 158 participants in Experiments 1, 2, and 3, the average InitPre duration was 1,388 ms (*SD* = 630 ms), whereas the average Init duration was 110 ms (*SD* = 31 ms). Thus, whereas we aimed for an ITD of 1,000 ms, for most trials (75.7 %) the actual ITD was slightly longer than the intended duration. In the supplementary material, we have provided participant-specific information on the durations of InitPre and Init.

## Online RT experiments using the QRTEngine

In order to demonstrate that typical chronometric effects can be observed when using the QRTEngine, we ran a number of classic behavioral RT experiments using this method. Accordingly, a Stroop task, an attentional blink task, and a masked-priming task were programmed using the QRTEngine and run online via AMT. Although standard Stroop and attentional blink effects have been observed using other online research tools as well, reliable negative masked-priming effects (cf. Eimer & Schlaghecken, 2002) have not been shown yet using JavaScript-based methods (Crump et al., 2013). The goal of the present experiments was to test whether the QRTEngine timing capabilities are sufficient to show the standard effects in these tasks.

## Experiment 1: Stroop task

The Stroop task is a classic paradigm that requires participants to identify the word color of congruent and incongruent color words. When word and word color are incongruent (e.g., the word “red” in green), RTs are slower and people make more errors than when the stimuli are congruent (MacLeod, 1991; Stroop, 1935).

### Method

#### Participants

The participants were recruited through AMT and were required to be located in the United States.^5^ Fifty-two participants completed the task: 29 were female, 43 were right-handed, and the average age was 35 years (*SD* = 12.54). Participants received financial compensation to complete the task, which lasted for approximately 5 min. Informed consent was given prior to the experiment. The ethics committee of the Leiden University Psychology section approved the experiment, as well as the following experiments described in this article.

#### Stroop task

The experiment was based partly on the Stroop task used by Crump et al. (2013). Participants completed 96 trials, of which 48 were congruent. The background color of the page was white, and words were presented in 50-point font in the colors red, green, blue, and yellow. Participants were asked to respond by typing the first letter of the color of the stimulus. The fixation point, word, and feedback were presented at the center of the page.

The trial started with a fixation cross displayed for 1,000 ms, which was followed by a blank screen displayed for 500 ms. Then the screen showing the color word was displayed until a response was made. Accuracy feedback was given after the response, using the word “CORRECT” or “INCORRECT” displayed for 500 ms in a black 30-point font.

#### Procedure

Participants found the task advertised as a HIT on AMT. They were informed that this HIT would require them to respond as accurately as possible and that it required full concentration. After participants had decided to take part, they were linked to our Qualtrics survey. This survey first collected some metadata, such as the browser version, operating system, and screen resolution used. Then, the survey estimated the speed of the participant’s Internet connection using the ITD estimation described earlier. When the connection speed was too low, the participant was kindly informed that he or she could not participate.

After participants had given informed consent, they were asked for their AMT worker ID in order that we could pay the participants later. This was followed by the instruction to maximize the browser window using F11 and reminding the participant that concentration was necessary for successful completion of the task. Following these general instructions, the specific instructions for the Stroop task were presented along with four examples. After this, participants started the task. When the task was completed, participants were asked for some demographic information before the survey ended.

### Results and discussion

We excluded four participants because of an accuracy below 80 %. A one-way repeated measures analysis of variance (ANOVA) on correct RTs and error rates was conducted with Congruency as the factor. For this experiment, as well as for the following experiments, a Greenhouse–Geisser correction was applied when the assumption of sphericity was violated. In these cases, we report corrected *p* values and uncorrected degrees of freedom. All significant effects (*p* < .05) are reported. The *MSE* and partial eta-squared are reported as measures of effect size.

**Fig. 2:**
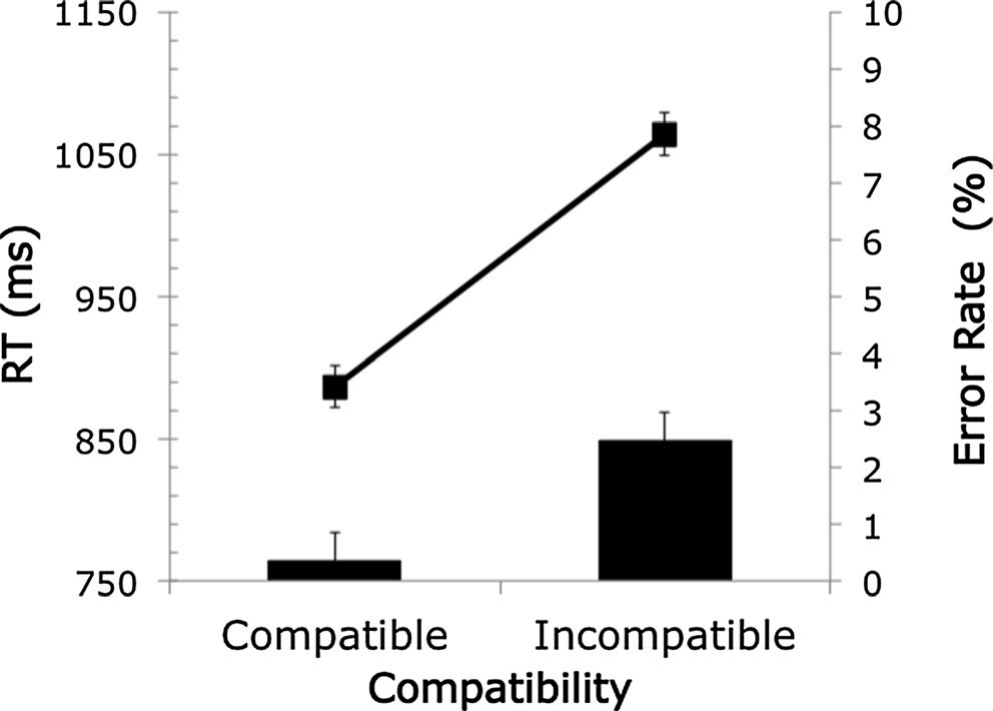
Mean reaction time (RT) and error rate for each condition in the Stroop task

Figure 2 shows the mean RT and error rate for each condition. In line with predictions, RTs were slower for incongruent (1,064 ms) than for congruent (887 ms) trials, showing a large Stroop effect (177 ms), *F*(1, 44) = 144.28, *MSE* = 4903, *p* < .001, η_p_^2^ = .766. Error rates were very low, with higher error rates for incongruent (2 %) than for congruent (0.3 %) items, *F*(1, 44) = 17.92, *MSE* = .001, *p* < .001, η_p_^2^ = .289. The observed RTs are consistent with those in the study by Crump et al. (2013), who reported 859 ms for congruent and 1,152 ms for incongruent trials.

## Experiment 2: Attentional blink

The attentional blink paradigm requires very precise control over the timing of stimulus presentation, and is therefore well suited to investigate the timing capabilities of the QRTEngine. Stimuli are presented in a rapid serial visual presentation (RSVP), and participants are asked to identify a first target (T1) and to decide whether a second target (T2) was present. The classic attentional blink effect shows that target identification of the T2 is impaired when it is presented 100–500 ms after T1 (Raymond, Shapiro, & Arnell, 1992). When T2 is presented directly after T1, there is less impairment, an effect called “lag 1 sparing.” When T2 is presented 2–8 places after T1, accuracy typically increases gradually with the distance from T1.

### Method

#### Participants

Participants were recruited through AMT and were required to be located in the United States. Among the 49 participants who completed the task, 20 were female, 37 were right-handed, and the average age was 34 years (*SD* = 12.54). Participants received financial compensation to complete the task, which lasted for approximately 5 min. Informed consent was taken prior to the experiment.

#### Attentional blink task

The experiment consisted of 80 trials and was partly based on the attentional blink task used by Crump et al. (2013). A gray 300-pixel square was displayed in the center of the page, the background was white, and stimuli were presented in the center of the square in a 50-point font. The letter sequences consisted of 7–14 pretarget letters and seven posttarget letters, all of which were uniquely selected from the whole alphabet and randomly ordered.^6^ T1 always appeared at the end of the pretarget sequence, and T2 was presented on 50 % of the trials at each of the posttarget positions. The present attentional blink experiment used black letters as distractors, a white letter as T1, and a black capital X as T2.

In each trial, a fixation cross was presented for 1,000 ms, followed by the stream of letters, each of which was presented for 100 ms. After the stream completed, participants were asked to identify T1 by pressing the corresponding letter on the keyboard. Then they were asked to press “1” if T2 had been present, or “0” if T2 had not. Both questions were displayed in a 15-point black font in the center of the gray square.

#### Procedure

The same procedure was used as described for Experiment 1.

### Results and discussion

**Fig. 3:**
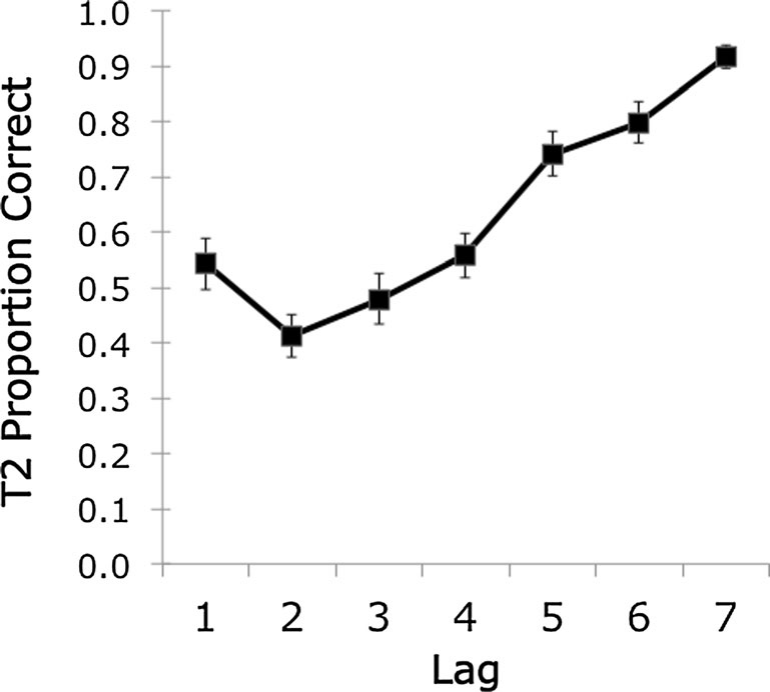
Mean Target 2 (T2) proportions correct as a function of T1–T2 lag

The analysis included only trials in which T1 was identified correctly. Figure 3 shows the mean proportions correct for detecting T2. A significant effect of lag is apparent, *F*(6, 252) = 31.89, *MSE* = .056, *p* < .001, η_p_^2^ = .432. The proportion correct is higher for lag 1 (.54) than for lag 2 (.41), *t*(42) = 2.82, *p* = .007, an effect demonstrating the typical lag 1 sparing. After lag 2, the proportion correct increases gradually to lag 7. In other words, the standard effects observed in attentional blink paradigms were reproduced in the present experiment. The proportions correct for the present experiment are quite similar to, although a little higher than, the results found by Crump et al. (2013), who reported a lag 1 proportion correct of .43 and a lag 2 proportion correct of .23. The differences between the lag 1 and lag 2 proportions correct in both studies are quite similar: .13 in our results, and .2 in the study by Crump et al. (2013).

## Experiment 3: Masked priming

The minimum duration for which a stimulus can be displayed on an average monitor is 16 ms, corresponding to a refresh rate of 60 Hz. Therefore, if the QRTEngine were proved capable of accurately presenting stimuli for 16 ms, that would indicate very good control over the timing. Masked-priming tasks are among the few paradigms that depend on such short presentation times. A classic result pattern of the masked-priming task developed by Eimer and Schlaghecken (2002) is a negative compatibility effect: When primes are presented for very short durations, a congruent prime increases the RT. We expected that a negative compatibility effect could indeed be found if the QRTEngine allowed very short stimulus presentation times.

### Method

#### Participants

Participants were recruited through AMT and were all located in the United States. Among the 57 participants who completed the task, 33 were female, 57 were right-handed, and the average age was 42 years (*SD* = 13.04). Participants received financial compensation to complete the task, which lasted for approximately 30 min. Informed consent was taken prior to the experiment.

#### Masked-priming task

The experiment consisted of 576 trials. The stimuli were black and measured 70 pixels in width and 40 pixels in height, and were presented in the center of a white page. There were six blocks of 96 trials; each block had a prime duration of 16, 32, 48, 64, 80, or 96 ms. The prime stimulus consisted of two arrows pointing is the same direction (<< or >>). The mask stimuli were chosen from 24 images consisting of eight randomly rotated and placed lines. The probe stimulus was similar to the prime.

#### Procedure

For this experiment, largely the same procedure was used as in Experiment 1. After the general instructions, participants received task-specific instructions and started the task. Each trial started with a fixation cross presented for 1,000 ms, followed by the prime with variable duration, which was followed by the mask presented for 96 ms. Next, a blank 48-ms interval was presented. Finally, the probe stimulus was presented for 96 ms and then removed immediately. Participants were instructed to respond as quickly and accurately as possible by pressing “S” when the probe consisted of left arrows, and “L” when the probe consisted of right arrows.

### Results and discussion

**Fig. 4:**
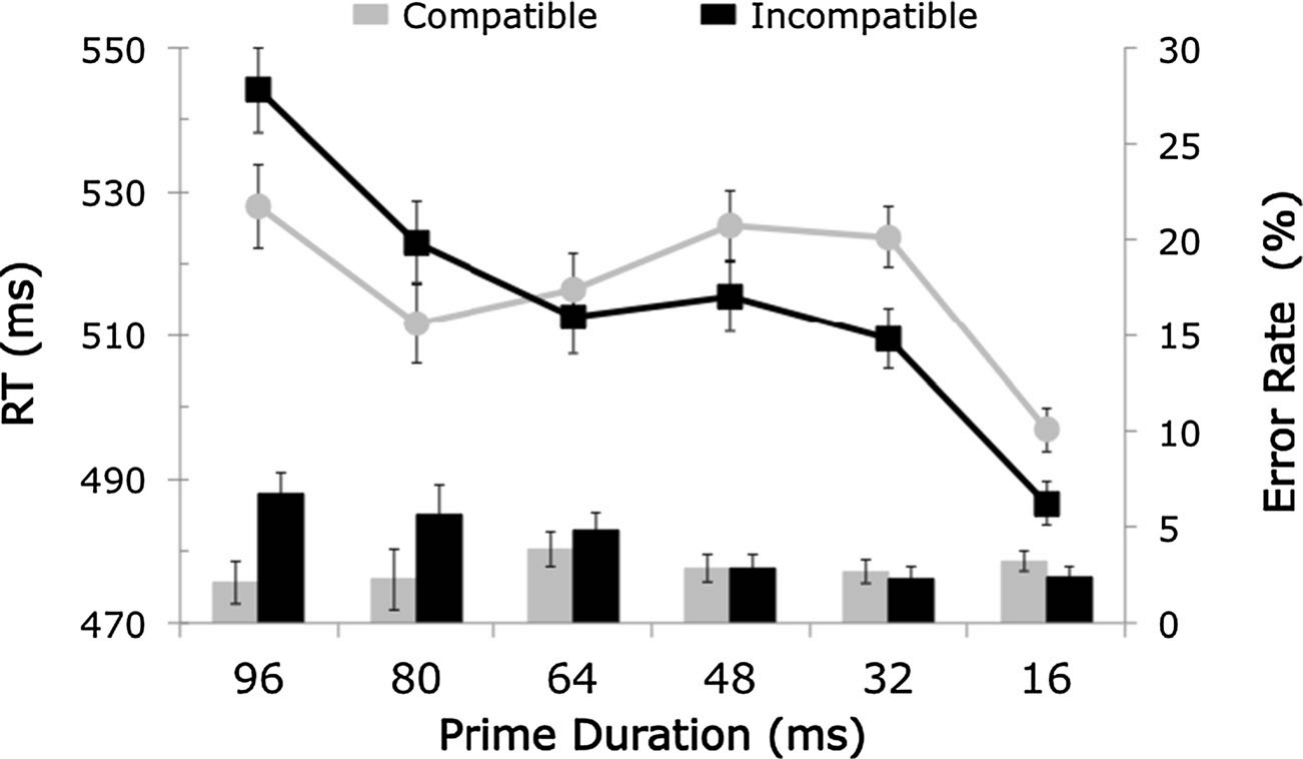
Mean reaction times (RTs) and error rates for each condition in the masked-priming task. Note the typical reversal of the compatibility effect for the shorter prime durations

Of the 57 participants, four outliers were omitted from analysis: three on the basis of low accuracy, one on the basis of RT (mean RT > 1,250 ms). Figure 4 shows the mean RTs and error rates for each of the prime durations. Importantly, the classic pattern of the masked-priming paradigm was reproduced: Relative to congruent primes, an incongruent prime increased RTs for longer prime durations (standard compatibility effect), whereas for shorter prime durations, the pattern reversed (negative compatibility effect). ANOVAs showed that the main effect of compatibility was not significant. The main effect of prime duration was significant, *F*(5, 260) = 5.49, *MSE* = 7875, *p* = .003, η_p_^2^ = .096, as was the interaction effect of compatibility and duration, *F*(5, 260) = 10.75, *MSE* = 549, *p* < .001, η_p_^2^ = .171. The compatibility effects were significant for all duration conditions except the 64-ms condition: for the 16-ms condition, compatibility effect = –10 ms, *t*(52) = 3.36, *p* = .001; for the 32-ms condition, compatibility effect = –14 ms, *t*(52) = 3.35, *p* = .002; for the 48-ms condition, compatibility effect = a marginally significant –10 ms, *t*(52) = 1.96, *p* = .056; for the 80-ms condition, compatibility effect = 11 ms, *t*(52) = –2.03, *p* = .047; for the 96-ms condition, compatibility effect = 16 ms, *t*(52) = –2.73, *p* = .009.

For the error rates, we found a significant main effect of prime duration, *F*(5, 260) = 3.4, *MSE* = .005, *p* = .031, η_p_^2^ = .061, a significant main effect of compatibility, *F*(1, 52) = 4.09, *MSE* = .007, *p* = .048, η_p_^2^ = .073, and a significant interaction effect, *F*(5, 260) = 7.57, *MSE* = .003, *p* < .001, η_p_^2^ = .127. The compatibility effects were significant for the 80-ms condition, compatibility effect = 3 %, *t*(52) = 2.14, *p* = .037, and for the 96-ms condition, compatibility effect = 5 %, *t*(52) = 4.21, *p* < .001.

The observed RTs are consistent with those from the study by Crump et al. (2013), who reported a compatibility effect of 34 ms in the 96-ms condition, whereas we found a compatibility effect of 16 ms in that condition. Since no negative compatibility effect was found in the study by Crump et al. (2013), we compared the negative compatibility effect with the one in the original masked-priming study by Eimer and Schlaghecken (2002). They reported a negative compatibility effect of about 12 ms in the 16-ms condition, very similar to the 10 ms from our results. Given that the pattern of our results is similar to the original findings by Eimer and Schlaghecken (2002), the results from the present experiment show that the QRTEngine can even be used to conduct behavioral experiments that rely on very short stimulus presentation timings.

## Summary of behavioral validation studies

By reproducing classic behavioral effects in three validation studies, we have provided important empirical validation for the QRTEngine. Although the Stroop and attentional blink effects had been found before in online studies (Crump et al., 2013), the present study, to our knowledge, was the first to provide results similar to the masked-priming effect originally reported by Eimer and Schlaghecken (2002) using JavaScript-based methods.

## Discussion

Performing behavioral research online is an interesting approach that is gaining increased popularity. The rapid development of Web-browser technology and the emergence of recruiting platforms such as AMT have facilitated this development. We think that the QRTEngine can help researchers conduct online behavioral research in an accessible and efficient way.

The QRTEngine provides a number of key features. First, the accuracy of its presentation timing was within ± 1 frame deviation in 97 % of the trials, which we observed by comparing the intended presentation times with a photodiode measurement. Further validation for its accurate timing was provided by an analysis of the behavioral results in typical RT paradigms run on AMT. A second key feature of the QRTEngine is that it works quite simply. In our experience, 60 min is often sufficient for a novice to follow the tutorial in the supplementary material to build a Stroop task. When using the method more often, building experiments will likely become very efficient, because elements can be easily exchanged within and between surveys. Because the experimental flow is determined by JavaScript snippets instead of by objects in a graphical user interface, trial procedures can also be implemented in a flexible manner. However, this flexibility also allows for programming the same experiment in different ways, possibly introducing small errors in the experimental design. We therefore recommend that users to share their experiments to facilitate research transparency and replicability (Asendorpf et al., 2013).

Although we believe that the QRTEngine indeed provides interesting benefits, potential users should be aware of some limitations. First of all, given that the observed accuracy of presentation timing is ± 1 frame deviation in 97 % of trials, the presentation timing capabilities of the QRTEngine are certainly not as good as those of software like E-Prime (Schneider et al., 2002) or comparable packages for offline research, since these solutions have considerably more control over the operating system. Furthermore, timing is partly dependent on the participant’s Web browser: For older browsers, the timing is less precise. Additionally, when using the time stamps logged during an experiment, it is important to keep in mind that these time audits are subject to measurement error. We expect that the limitations regarding timing will gradually be resolved in the future, since Web browsers develop very rapidly with a focus on speed. Although our validation studies suggest that the QRTEngine is often capable of providing very short presentation times of up to one frame (i.e., 16.7 ms when using a 60-Hz refresh rate), inaccuracy will always remain when running online experiments directly in the browser, especially with very short presentation times. Considering these validations, it should be noted that we did not perform any validation on mobile devices such as tablets or smartphones. Given these considerations, online studies will introduce considerable nonsystematic noise in terms of timing, which might affect the sensitivity to small effects in RTs. In extreme cases, measurement error could therefore be speculated to become even twice as high as in typical lab experiments. Increasing the sample size is the easiest way to reduce the impact of this problem. As a very conservative rule of thumb, researchers might therefore consider multiplying their sample size, based on conventional power analyses of lab studies, by a factor 4 in order to be equally sensitive to the similar effects observed in the lab.

Second, the ITD may occasionally exceed the desired duration between trials. This is because the method is dependent on Qualtrics servers and the Internet connection of the participant. We therefore recommend not using the QRTEngine when it is necessary for the intertrial interval to be exactly consistent or when the intertrial interval needs to be very short. Using a test that measures the Internet connection quality before the start of an experiment will partly help to reduce the impact of this limitation.

Third, at present the QRTEngine allows for presenting text and pictures only, so studies requiring auditory and movie stimuli are not supported. Furthermore, researchers should also be aware that designs in which the types of trials cannot be specified in advance in Loop & Merge lists might be difficult to implement in Qualtrics. Besides these QRTEngine-specific recommendations, we would remind potential users of the caveats associated with online RT experiments in general, which we discussed in the introduction.

The growing interest in online behavioral research has led to numerous studies regarding the benefits and validity of such results, mostly focusing on data collected using AMT (Buhrmester et al., 2011; Germine et al., 2012; Paolacci et al., 2010). Although the QRTEngine can be used to conduct online research through any channel, it provides the additional benefit of being JavaScript-based, which complies with AMT’s regulations that workers should not be asked to download additional software. Conducting online behavioral research provides numerous advantages and is starting to emerge as a serious alternative for many researchers. We think that the findings described here show that the QRTEngine may provide a valuable tool for conducting such research.

## Electronic supplementary material

Below is the link to the electronic supplementary material.

ESM 1 (PDF 1.01 mb)

## Footnotes

1 During publication of this article we learned about an alternative JavaScript method that is worth mentioning: jsPsych (de Leeuw, 2014). We provide a comparison of QRTEngine and jsPsych, created in collaboration with the jsPsych author, on www.qrtengine.comm

2 On the basis of the StatCounter browser-use statistics for June 2014 (http://caniuse.com/usage_table.php, retrieved July 1, 2014), 85 % of currently used desktop browsers support rAF. For a complete overview of rAF support, including previous browser versions, see http://caniuse.com/requestanimationframe. Please note that these statistics are not absolute; as the site states, “a feature is considered ‘supported’ when its primary purpose is largely fulfilled.” Therefore, QRTEngine logs rAF and timer support for every participant.

3 For a complete overview of support, including previous browser versions, see http://caniuse.com/high-resolution-time.

4 Participation was only allowed and data were only acquired when the mean +2 standard deviations of an individual’s InitPre did not exceed 2,000 ms. These InitPre’s were measured in a separate question block, which looped ten trials while showing an initialization progress bar, before the experiment started.

5 Although these Mechanical Turk participants had a registered location within the United States, we cannot exclude the possibility that some participants were actually performing the experiment from another country.

6 We aimed to present eight posttarget positions, but due to a programming error, the eighth position was never presented. We therefore do not include the lag 8 condition in the analyses.
