# Supplementary material for: QRTEngine: An easy solution for running online reaction time experiments using Qualtrics
Source: Behav Res Methods. 2014 Nov 19;47(4):918–29. doi: 10.3758/s13428-014-0530-7 (PMC4636512; doi:10.3758/s13428-014-0530-7)
Supplement: Supplementary file 1 — (PDF 1.01 mb) [file 13428_2014_530_MOESM1_ESM.pdf]

Supplementary materials for:

QRTEngine: An easy solution for running online reaction time experiments using Qualtrics

Jonathan S. Barnhoorn[1, 2, 3], Erwin Haasnoot[1,4], Bruno R. Bocanegra[1,5] and Henk van Steenbergen[1,5]

[1] *Leiden University, Institute of Psychology, Wassenaarseweg 52, Leiden, 2333AK, The Netherlands*
[2] *Department of Cognitive Psychology and Ergonomics, Faculty of Behavioral, Management and Social Sciences, University of Twente, The Netherlands*
[3] *MIRA Research Institute, University of Twente, The Netherlands*
[4] *University of Sheffield, UK*
[5] *Leiden Institute for Brain and Cognition*

jonathanbarnhoorn@gmail.com. erwinhaasnoot@gmail.com. {b.r.bocanegra. hvansteenbergen}@fsw.leidenuniv.nl

Corresponding author:
Dr. Henk van Steenbergen
Wassenaarseweg 52
2333 AK Leiden
The Netherlands
Phone number: +31 (0)71 527 3655
Fax number: +31 (0)71 527 3783
E-mail address: HvanSteenbergen@fsw.leidenuniv.nl

Supplementary material in this document

PART A. Manual for building a Stroop task.

PART B. Calculated, intended and diode measured duration for each load-condition, divided per system.

PART C. Diode validation results: tabulated summary.

PART D. Participant-specific InitPre and Init calculated duration results.

The following material can be found on www.qrtengine.com

- The QRTEngine JavaScript code
- The Excel file with the trial-list for the Stroop task

PART A. USING THE QRTENGINE

In this tutorial we describe how to build a Stroop (MacLeod, 1991; Stroop, 1935) task using the QRTEngine. The task will consist of one type of trial containing three screens: a fixation cross displayed for 1000 ms; a blank screen displayed for 500 ms, and a screen showing a color word with congruent and incongruent ink colors that is displayed until a response is made. Participants are instructed to respond to the ink color of the word by typing the first letter of the corresponding color (e.g. "r" if the ink color is red). In order to build this task, one only needs a computer with Internet connection, and a Qualtrics account.

Step 1: Setting up the survey

The Stroop RT task will be part of a survey, so the first step is to create a new survey. After creating a new survey, the QRTEngine needs to be included. To do this, go to *Look & Feel* > *Advanced* and paste the QRTEngine JavaScript code (that is freely available on www.qrtengine.com) in the 'Header' field. Click *Save*. The QRTEngine is now available for use in the survey.

Before the QRTEngine is able to work, one needs to set a number of embedded data fields. These are essentially global variables that the QRTEngine needs to be able to use when the survey is running. Click *Survey Flow* > *Add a New Element Here* > *Embedded Data* and add the fields as displayed in Figure 1A, then click *Save*.

A final step in setting up the survey is selecting the right layout. Although aspects of the layout can be manually manipulated using CSS, one can typically use the standard Qualtrics 'Minimal' layout. This layout uses a plain white background with black text and can be found under the *Look & Feel* tab. After setting the layout, the QRTEngine is included successfully in the survey.

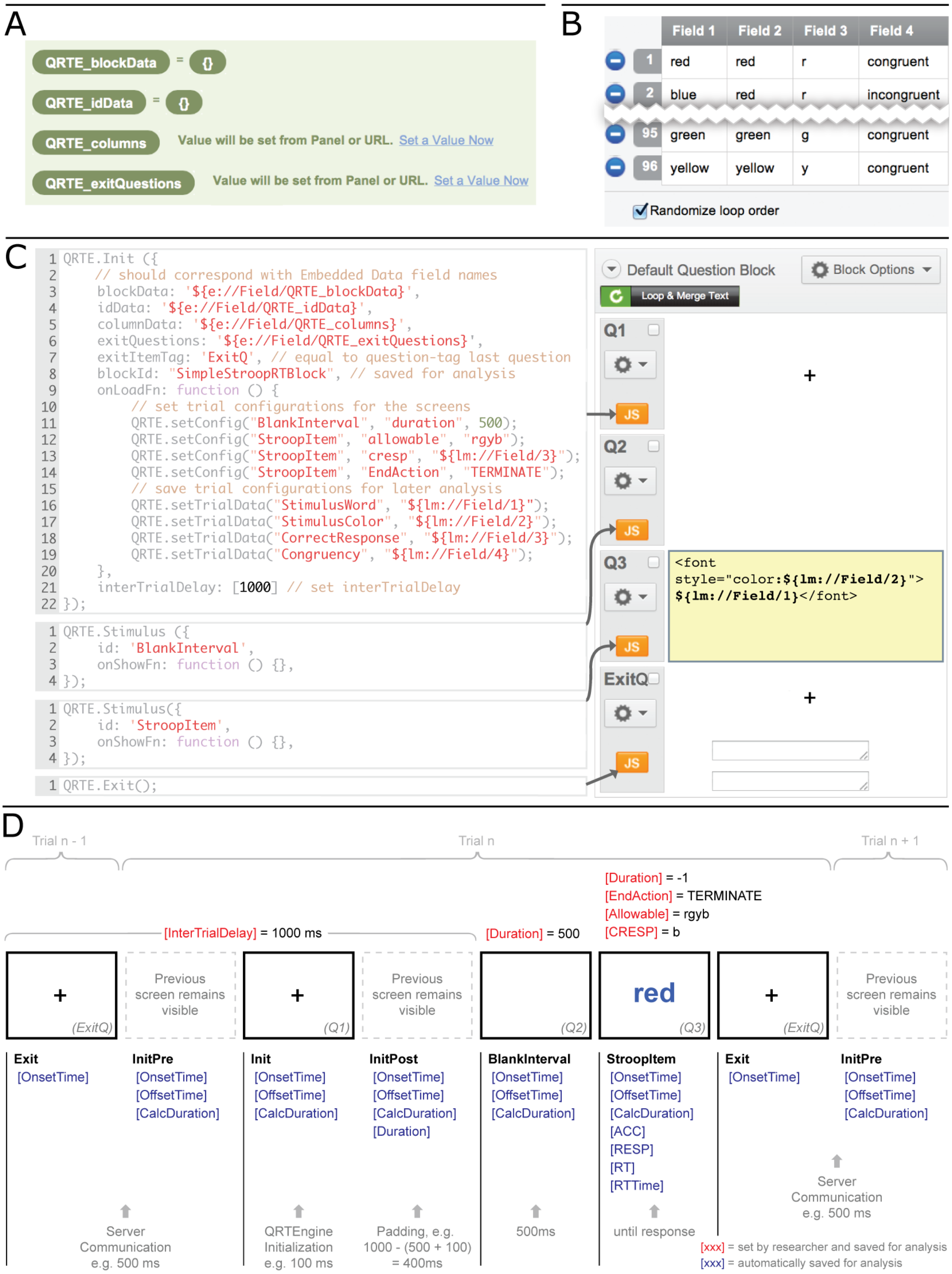

Figure 1. A) Screenshot of what the embedded data overview should look like for the Stroop RT task. B) A screenshot of the Loop & Merge list. In the Stroop RT task 96 trials will be displayed, 4 columns are needed to define the variable content for each trial. C) A screenshot of the question block along with the JavaScript for each question. Each question represents a screen in the task. D) A schematic overview of a trial in the Stroop RT task.

Step 2: Setting up the list of trials

On the ‘Edit Survey’ page, an empty question block is presented. This question block will be used to present trials from the Stroop task. We start by defining the list of trials to be displayed using the Qualtrics Loop & Merge (L&M) functionality. L&M essentially loops a question block, providing different properties (in our case, colors, color words and correct responses) for each trial. To turn on the L&M functionality for a question block: click on *Block options* > *Loop & Merge* > *Turn On Loop & Merge*. Here, the list of trials can be created by adding as many columns and rows as necessary (it is often helpful to paste a list from a spreadsheet editor like Excel). An example of a list for the Stroop task is shown in Figure 1B. The first column defines the word that will be displayed. The second column defines the ink color in which the word will be displayed. The third column defines the correct response key. The fourth column indicates whether the ink color is congruent with the word that is shown. This allows us to store this variable for later data analysis using JavaScript snippets (see below). Finally, we check the option *Randomize Loop Order* to ensure that the trials are presented in random order.

Step 3: Setting up the trial

Next, we add the screens showing the content of a trial. In the question block, click on *Create a New Item*, a new question of the type ‘Multiple Choice’ is inserted. The new question contains three bullets, these will not be displayed or used, and can be removed. Add more questions until there are four questions. Select the last question and change the *Item Type* to ‘Text Entry’, the *Text Type* to ‘Form’ and set the number of *Form Fields* to two. The QRTEngine will use these fields for data storage. The

form fields will not be visible when running the experiment. For the first and the last questions, add a '+' sign that will function as a fixation cross.

To present the stimulus, use the *HTML View* mode to insert the HTML code displayed in Figure 1C in question 3. Note that the values from the first two columns of the L&M list are used for the stimulus (in Q3) using the "Piping text" feature. Accordingly, the code `${lm://Field/2}` will insert the color from the second column, whereas the code `${lm://Field/1}` will insert the word from the first column.

Finally, change the question tag of the last question to 'ExitQ'. As long as the survey question tags (in the figure: Q1; Q2: Q3; and ExitQ) are unique in the survey the exact naming is arbitrary. However, it is important to correctly refer (in the JS snippet) to the tag that belongs to the final question as will be explained later.

The question block now should look like the one depicted in Figure 1C. Note that each trial consists of a fixation cross, a blank screen, and a screen showing the color word stimulus. However, while we want to display only three screens, we have added four questions. This is to allow for accurate timing across trials: the engine assumes that the stimulus showed during the inter-trial interval is available both at the first and the last position of the question block.

Step 4: Adding the timing

As a final step, some JavaScript code is added to each question to set the timing features of the trial. Select the question, then click the gear-icon on the left of the question and click '*Add JavaScript*'. For each question, replace the standard JavaScript Qualtrics has added with the code that is displayed in Figure 1C. In the code, the lines containing `setConfig` set the configurations for the screens, like the duration of the 'BlankInterval' screen and the allowable responses of the 'StroopItem'

screen. Because the content of the L&M list is not stored automatically, the lines containing `setTrialData` determine which values from the L&M list will be saved for later data analysis.

Note that the JavaScript snippet for the first question also contains the following line:

```
exitItemTag: 'ExitQ',
```

The value mentioned here should correspond to the name of the question tag of the final question of the current question block (“ExitQ” in our example).

Step 5: Run the survey

If all the elements and code-snippets have been added the Stroop RT task should work properly. To test the survey, press the ‘*Preview Survey*’ button and run the task. To start distributing the survey (for instance, via AMT), one can simply click the *Launch Survey* button or the *Distribute* Survey tab.

Importantly, this RT task now does not include instruction screens and other tasks. Those can be added easily using new question blocks. Note that when the engine is included in a survey, all content including the ‘next’ button is hidden automatically for all questions. To unhide this content, add the following JavaScript snippet to a question:

```
Qualtrics.SurveyEngine.addOnload(function()
{
  QRTE.unhideQuestions();
  $('NextButton').style.display = 'inline';
});
```

Step 6: Exporting and analyzing participant data

The data that has been collected using the survey can be found under the '*View Results*' tab. Because Qualtrics uses a standard wide format (each participant's data is stored in a single row), all trial information is stored using JSON representation. A data parser has been published (freely available on www.qrtengine.com) that allows to convert and export a Qualtrics QRTEngine CSV file to a long format (each row presents data of a single trial). The QRTEParser is a Java program that can be used on different platforms (e.g., Windows, OSX, and Linux). Standard packages such as SPSS and Excel can then be used to read the resulting CSV data file and perform data aggregation and statistics.

PART B. CALCULATED, INTENDED AND DIODE MEASURED DURATION FOR EACH LOAD-CONDITION, DIVIDED PER SYSTEM

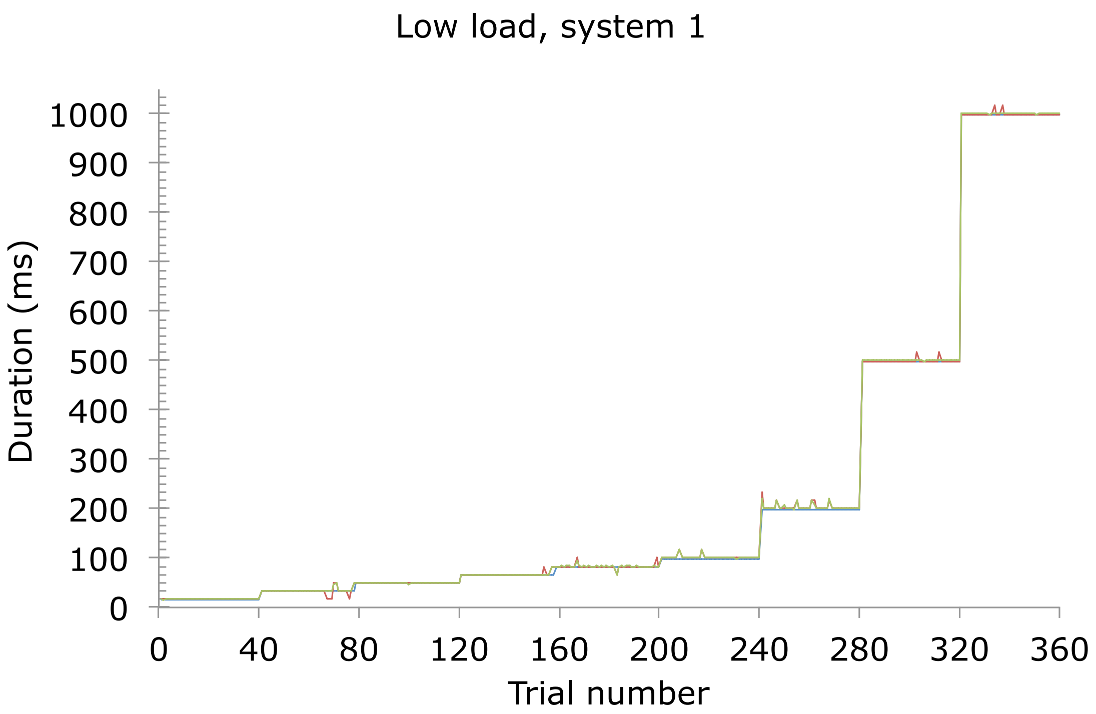

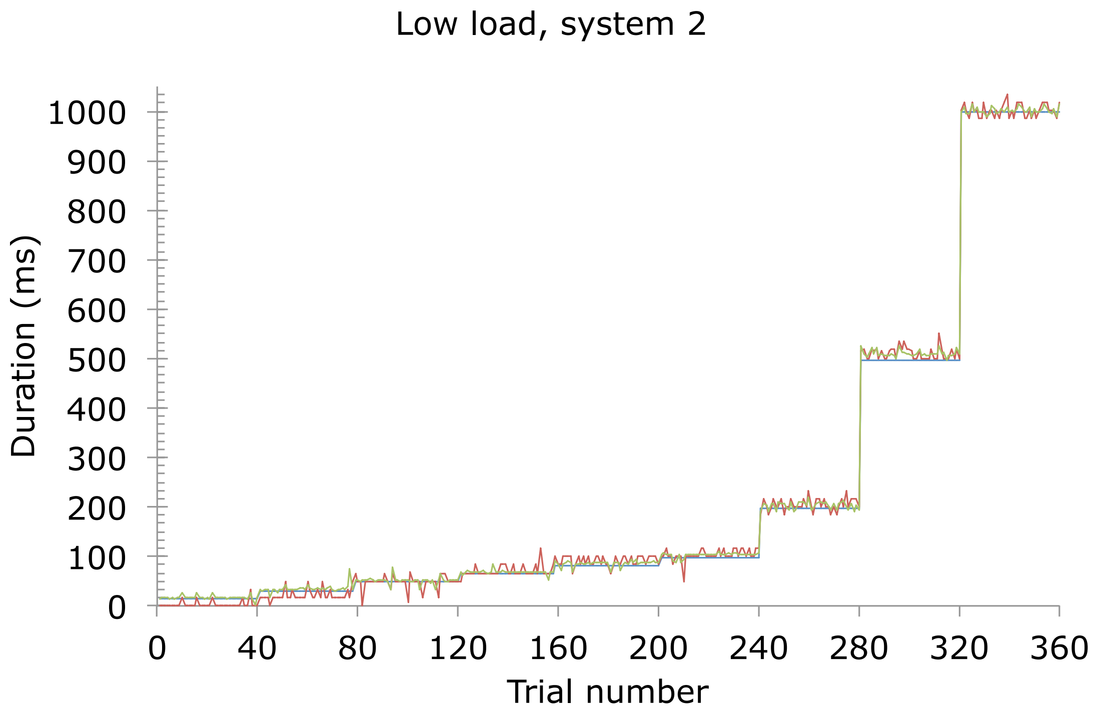

Figure S1. Calculated (green), intended (blue) and diode measured (red) durations over the 360 trials for the low-load conditions (milliseconds).

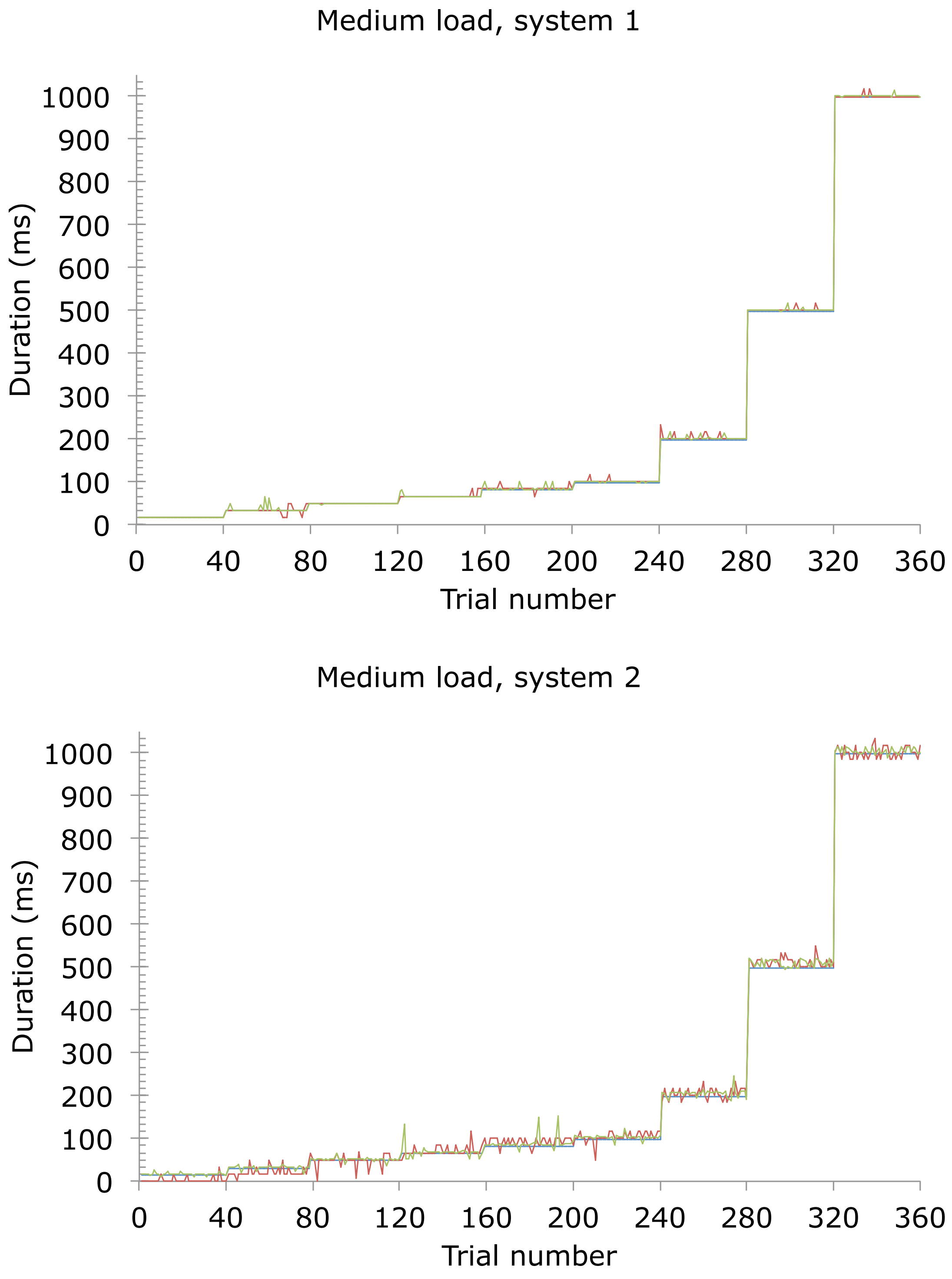

Figure S2. Calculated (green), intended (blue) and diode measured (red) durations over the 360 trials for the medium-load conditions (milliseconds).

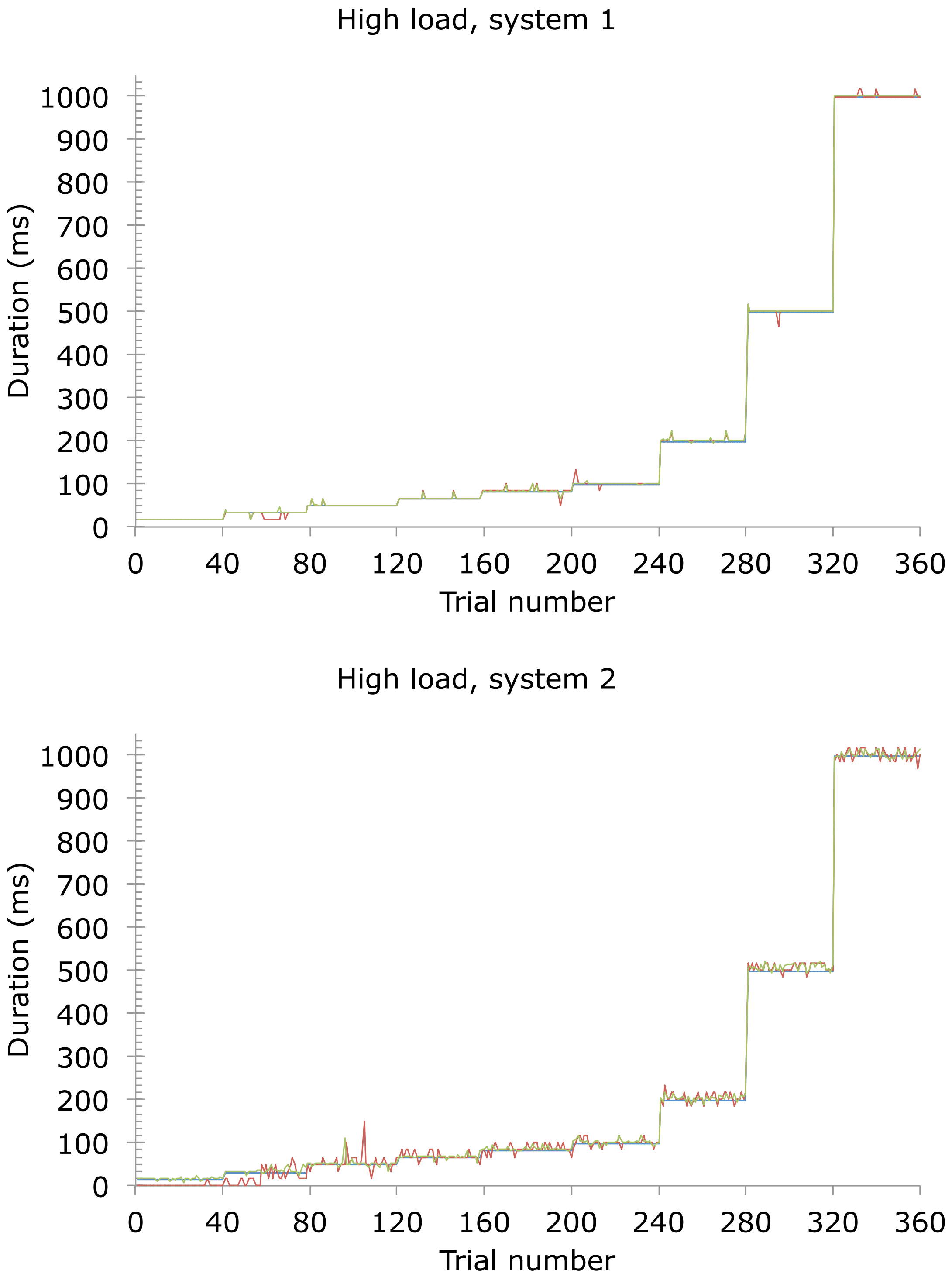

Figure S3. Calculated (green), intended (blue) and diode measured (red) durations over the 360 trials for the high-load conditions (milliseconds).

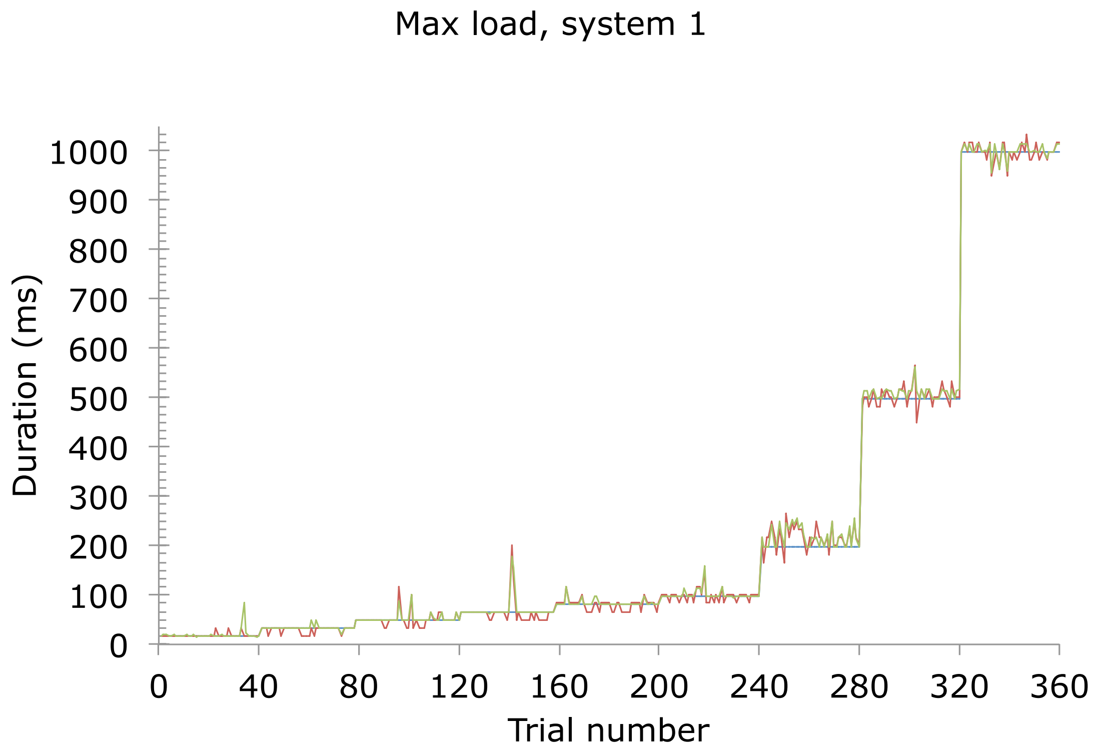

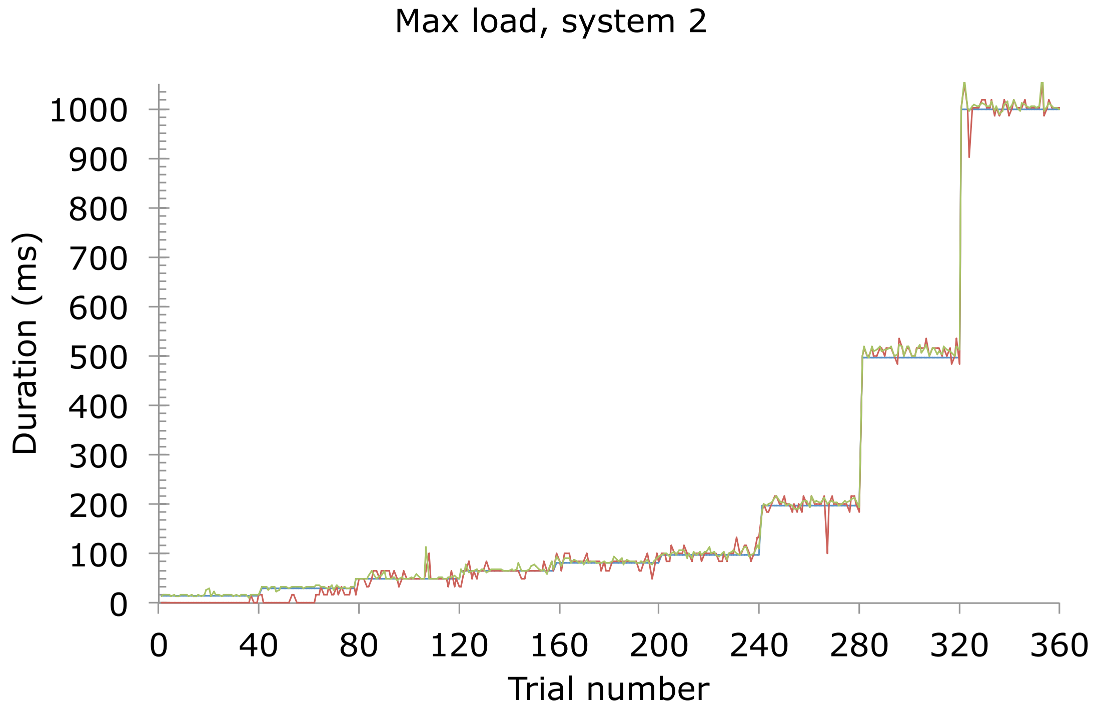

Figure S4. Calculated (green), intended (blue) and diode measured (red) durations over the 360 trials for the maximum-load conditions (milliseconds).

PART C. DIODE VALIDATION RESULTS SUMMARY

Table S1. Average absolute difference between calculated and diode measured duration (milliseconds).

Load	*System 1*	*System 2*	*Mean*
Low	0.9	9.7	*5.3*
Med	1.8	9.6	*5.7*
High	1.4	10.1	*5.8*
Max	6.6	10.0	*8.3*
	2.7	*9.9*	*6.3*

Table S2. Percentage of trials in which the calculated and diode measured duration did not match.

Load	*System 1*	*System 2*	*Mean*
Low	3.1%	43.9%	*23.5%*
Med	8.1%	40.6%	*24.3%*
High	4.7%	46.1%	*25.4%*
Max	33.1%	40.3%	*36.7%*
	12.2%	*42.7%*	*27.5%*

Table S3. Average absolute difference between intended and diode measured duration (milliseconds).

Load	*System 1*	*System 2*	*Mean*
Low	1.2	9.9	*5.5*
Med	2.2	10.9	*6.6*
High	1.5	9.5	*5.5*
Max	9.3	10.7	*10.0*
	3.5	*10.2*	*6.9*

Table S4. Percentage of trials in which the intended and diode measured duration did not match.

Load	*System 1*	*System 2*	*Mean*
Low	6.9%	54.2%	*30.6%*
Med	12.8%	54.2%	*33.5%*
High	8.1%	50.8%	*29.4%*
Max	40.8%	51.4%	*46.1%*
	17.2%	*52.6%*	*34.9%*

Table S5. Percentage of trials in which the intended and diode measured duration differed by 0, 1, 2, or more than 2 frames. In the experiment, 10 different stimulus presentation durations were used: the following table presents the combined results of these 10 conditions.

	System 1				System 2			
Frames deviation	0	1	2	> 2	0	1	2	> 2
Low load	93.0 %	6.6 %	0.2 %	0.0 %	46.1 %	50.5 %	2.2 %	1.1 %
Med load	87.2 %	12.2 %	0.5 %	0.0 %	45.8 %	45.8 %	6.9 %	1.3 %
High load	91.9 %	7.2 %	0.8 %	0.0 %	49.1 %	46.3 %	3.8 %	0.5 %
Max load	59.1 %	32.7 %	3.6 %	4.4 %	48.6 %	42.2 %	7.7 %	1.3 %

Table S6. Percentage of trials in which the intended and diode measured duration differed by 0, 1, 2, or more than 2 frames. In the experiment, 10 different stimulus presentation durations were used: the following table presents the results of trials were only the stimulus was presented for the duration of 1 frame (16.67 ms).

	System 1				System 2			
Frames deviation	0	1	2	> 2	0	1	2	> 2
Low load	100 %	0.0 %	0.0 %	0.0 %	10.0 %	90.0 %	0.0 %	0.0 %
Med load	95 %	5.0 %	0.0 %	0.0 %	7.5 %	92.5 %	0.0 %	0.0 %
High load	100 %	0.0 %	0.0 %	0.0 %	2.5 %	97.5 %	0.0 %	0.0 %
Max load	92.5 %	7.5 %	0.0 %	0.0 %	5.0 %	95.0 %	0.0 %	0.0 %

Table S7. Percentage of trials in which the intended and diode measured duration differed by 0, 1, 2, or more than 2 frames. In the experiment, 10 different stimulus presentation durations were used: the following table presents the results of trials were only the stimulus was presented for the duration of 2 frames (33.33 ms)

	System 1				System 2			
Frames deviation	0	1	2	> 2	0	1	2	> 2
Low load	81.6 %	18.4 %	0.0 %	0.0 %	13.2 %	84.2 %	2.6 %	0.0 %
Med load	63.1 %	31.6 %	5.3 %	0.0 %	2.6 %	42.1 %	55.3 %	0.0 %
High load	73.7 %	26.3 %	0.0 %	0.0 %	13.1 %	57.9 %	29.0 %	0.0 %
Max load	78.9 %	21.1 %	0.0 %	0.0 %	0.0 %	50.0 %	50.0 %	0.0 %

PART D. INITPRE AND INIT CALCULATED DURATION RESULTS.

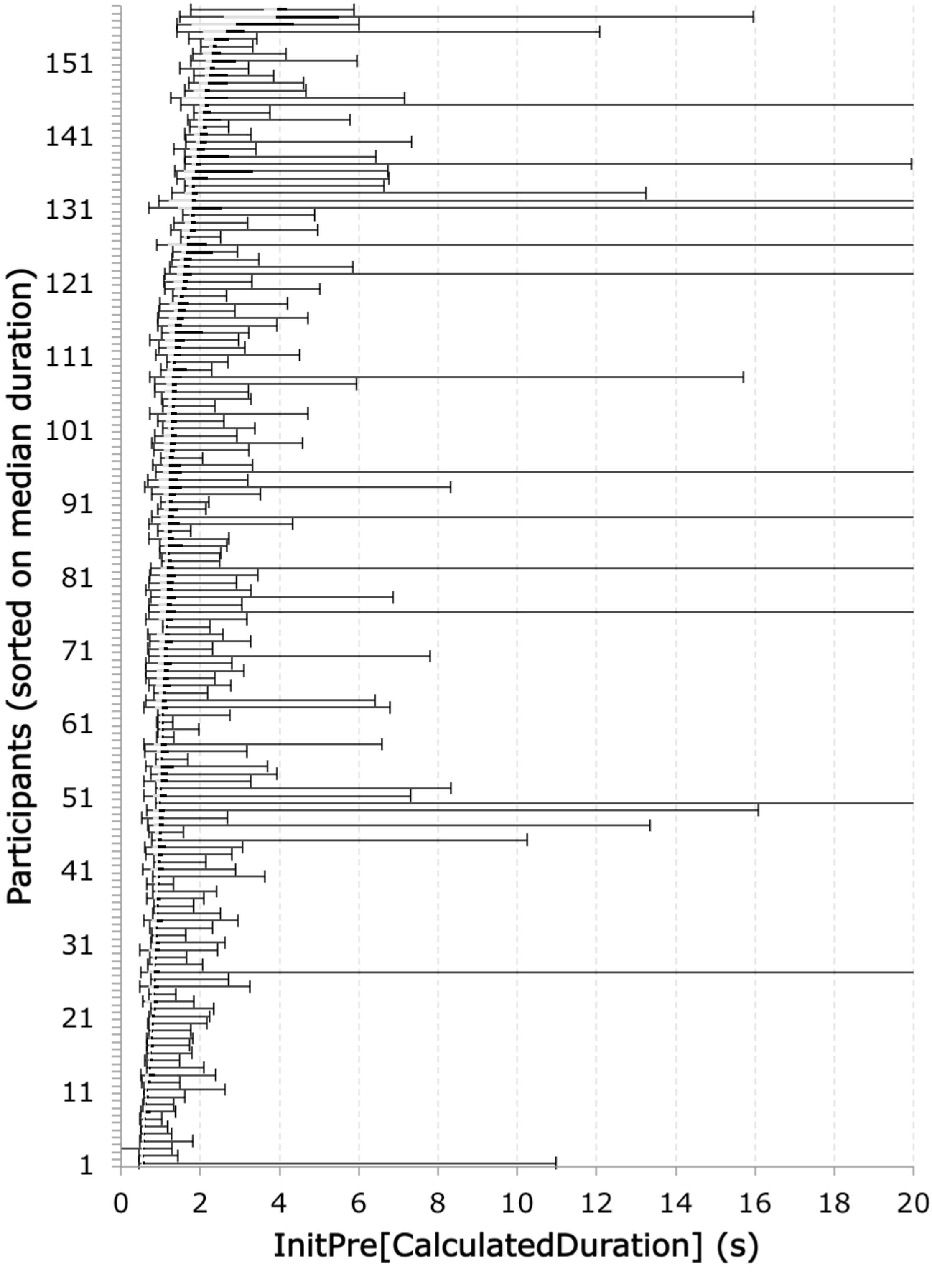

Figure S5. Overview of calculated durations per participant of the InitPre period (during which server calculation takes place) as measured over the three validation studies. For these durations, the average mean = 1388 ms; average median = 1276 ms; average standard deviation = 630 ms; average minimum = 915 ms; average maximum = 7174 ms; average 25th percentile = 1134 ms; average 75th percentile = 1469 ms.

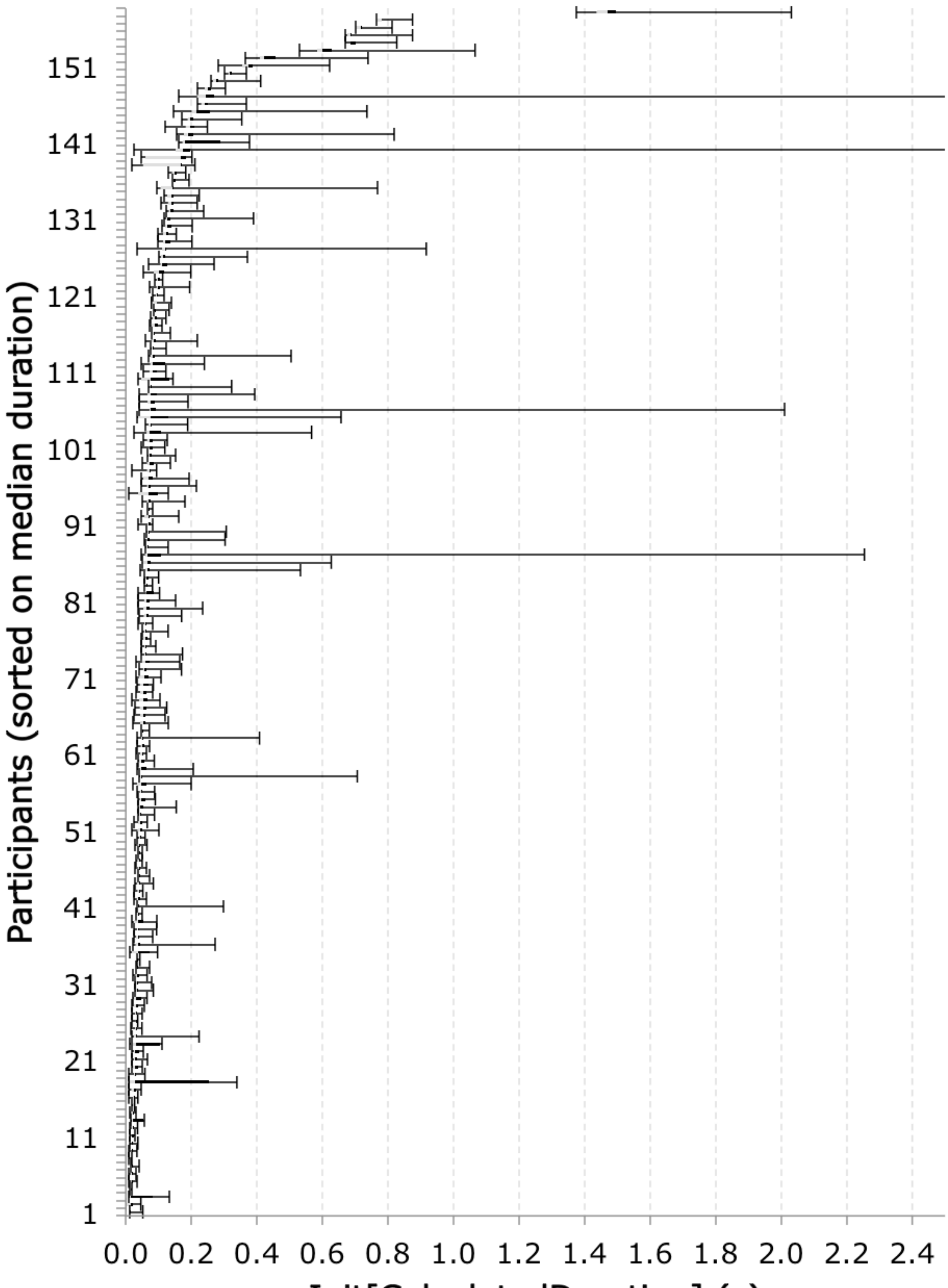

Figure S6. Overview of calculated durations per participant of the Init period (during which the QRTEngine is initialized at the start of each trial) as measured over the three validation studies. For these durations, average mean = 110 ms; average median = 105 ms; average standard deviation = 31 ms; average minimum = 83 ms; average maximum = 410 ms; average 25th percentile = 97 ms; average 75th percentile = 117 ms.
